# T-Allele Carriers of Mono Carboxylate Transporter One Gene Polymorphism rs1049434 Demonstrate Altered Substrate Metabolization during Exhaustive Exercise

**DOI:** 10.3390/genes15070918

**Published:** 2024-07-14

**Authors:** Benedikt Gasser, Alain Dössegger, Marie-Noëlle Giraud, Martin Flück

**Affiliations:** 1 Department of Sport, Physical Activity and Health, University of Basel, 4001 Basel, Switzerland; benedikt.gasser@yahoo.com (B.G.); alain.doessegger@baspo.admin.ch (A.D.); 2 Swiss Federal Institute of Sport Magglingen SFISM, 2532 Magglingen, Switzerland; 3 Cardiology, Department of Medicine, University of Fribourg, 1700 Fribourg, Switzerland; marie-noelle.giraud@unifr.ch; 4 Institute for Biomedical Research into Human Movement and Health, Manchester Metropolitan University, Manchester, UK

**Keywords:** lactate, ketone, exercise, skeletal muscle, muscle fiber, slow, aerobic, metabolism, fatigue, mass spectroscopy, genotype

## Abstract

Background: Polymorphism rs1049434 characterizes the nonsynonymous exchange of adenosine (A) by thymidine (T) in the gene for monocarboxylate transporter 1 (*MCT1*). We tested whether T-allele carriers of rs1049434 demonstrate increased accumulation of markers of metabolic strain. Methods: Physically active, healthy, young male subjects (n = 22) conducted a power-matched one-legged cycling exercise to exhaustion. Metabolic substrates in capillary blood, selected metabolic compounds, and indices for the slow oxidative phenotype of vastus lateralis muscle were quantified in samples collected before and after exercise. The genotypes of the rs1049434 polymorphism were determined with polymerase chain reactions. Results: One-legged exercise affected the concentration of muscle metabolites entering the tricarboxylic acid cycle, such as acetyl-co-enzyme A (+448%) and acetyl-L-carnitine (+548%), muscle glycogen (−59%), and adenosine monophosphate (−39%), 30 min post-exercise. Exercise-related variability in the muscular concentration of glycogen, long-chain acyl co-enzyme As and a triglyceride, nicotinamide adenine dinucleotide (NADH), and adenosine monophosphate (AMP) interacted with rs1049434. T-allele carriers demonstrated a 39% lesser reduction in glycogen after exercise than non-carriers when NADH increased only in the non-carriers. Muscle lactate concentration was 150% higher, blood triacyl-glyceride concentration was 53% lower, and slow fiber percentage was 20% lower in T-allele carriers. Discussion: The observations suggest a higher anaerobic glycolytic strain during exhaustive exercise and a lowered lipid handling in T-allele non-carriers.

## 1. Introduction

Human performance is set by anatomical design, whereby a considerable genetic contribution has been identified in the expression of traits in endurance performance (reviewed in [1,2,3]), especially in response to training [4,5,6]. Recently, sequence variants, commonly referred to as polymorphisms, in several genes have been found to influence human performance variability by affecting metabolic and contractile functions of the cardiopulmonary system and skeletal muscle. Therefore, more than 100 gene polymorphisms have been associated with athlete status [7,8] and show a well-established, physiologically explainable basis. Further deepening the understanding of the gene–environmental mechanisms might support training descriptions for both healthy and trained individuals of all ages.

As is well known from metabolic studies on short maximal physical activities to exhaustion, the strain on the aerobic system can be considerable (reviewed in [9]). This is emphasized, for example, by the effects of sprinting exercises lasting up to one minute on the lactate concentration. During short-term exercise, the concentration of the end product of anaerobic glycolysis, lactate, in the blood can increase from values close to 1 mmol/L to over 12–14 mmol/L at maximum exertion [10,11]. Therefore, considerable inter-individual variability can be observed, which relates to the intensity of exercise [12], as well as the capacity of biochemical pathways that determine the rate of local aerobic metabolism [11,13] and efflux rates of lactate from skeletal muscle [14]. In this respect, the proton-linked monocarboxylate transporter 1 (MCT1, also solute carrier family 16 member 1) plays a crucial role in the transport of lactate from lactate-producing organelles to lactate-consuming organelles [15,16]. In skeletal muscle, MCT1 is present in the plasma membranes of muscle fibers (sarcolemma) and erythrocytes [16,17]. In addition, MCT1 is also involved in the transmembrane transport of other monocarboxylates, such as pyruvate, acetate, and the ketone bodies acetoacetate and β-hydroxybutyrate (bHb) [18], whose concentrations change with exercise and may serve as an alternative fuel source for skeletal muscle [19]. There seems to be an association between MCT1 and the differentiation of muscle metabolism and muscle fiber type [20]. The rates of MCT1-mediated lactate transport are subject to regulation by physical activity levels and genetic influences.

For instance, exercise seems to increase the MCT1 content [16,21], which may also affect the MCT1 content in erythrocytes [17]. Furthermore, a prominent gene polymorphism, rs1049434, is associated with differences in lactate transport rates in erythrocytes and human skeletal muscle [22]. Rs1049434 describes a nucleotide exchange of adenosine (A) for thymidine (T) at position 1470, which results in the exchange of glutamic for aspartic acid at amino acid position 490 and reduces lactate transport rates by 60–65% [23]. It has been demonstrated that lower rates of lactate accumulation exist in resistance-trained and endurance-trained homozygous carriers of the A-allele than in T-allele carriers during exercise to exhaustion [23,24,25,26]. The functional relevance of this respective influence on blood lactate accumulation with intense exercise is supported by observations that the *MCT1*-rs1049434 genotype is related to different physical performance phenotypes. Thereby, the A-allele is more frequently observed in elite endurance athletes compared to those specializing in sprint/power sports [22,23,25,26,27,28,29]. This indicates that the *MCT1* A-allele might be associated with better endurance performance due to enhanced lactate transport from producing muscle fibers via the blood to slow-twitch oxidative muscle fibers that can use lactate as fuel, thereby increasing endurance capacity [18]. The metabolism of lactate relies on the concentrations of the reduction equivalent, the reduced form of nicotinamide adenine dinucleotide (NADH). NADH, in turn, sets the rate at which ATP (adenosine triphosphate) may be produced in mitochondria via a coupling to complex I of the mitochondrial respiration chain [30]. The ratio between the reduced and oxidized forms of nicotinamide has recently also been recognized to affect the activity of glycolytic metabolism [31]. To the best of our knowledge, whether differences in the accumulation of lactate in skeletal muscle tissue with exhaustive exercise exist between the rs1049434 genotypes, and the extent to which this involves differences in ketone bodies and metabolites that relate via the reduction equivalent of NADH to the activity of glycolysis and TCA-mediated charging of high-energy phosphates, as well as lipidic substrates for general metabolism, has not been elucidated [19]. Therefore, we aimed to add knowledge on the physiologically explainable associations between different alleles of rs1049434 and lactate, ketone bodies, NADH, as well as lipidic substrates in working muscles. We hypothesized that there is an effect of rs1049434 gene polymorphisms on exercise-modulated metabolism of lactate, ketones, and metabolic pathways that are under the feed-forward and feed-back influence of NADH, such as the TCA-cycle-mediated charging of high-energy phosphates, adenosine triphosphate and creatine phosphate (reviewed in [32]), and glycolytic flux. In addition, we assumed that all these factors are related to the proportions of the slow oxidative muscle phenotype in working muscle.

## 2. Materials and Methods

Subjects: Twenty-two healthy, male, white Caucasian subjects (27.0 ± 6.6 years; BMI: 23.8 ± 3.2 kg m^−2^) were recruited from white British men of the Greater Manchester Area. The exclusion criteria were smoking status, long-term poor health, age younger than 18 years or older than 40, and a relative VO_2_max less than 40 mL O_2_ min^−1^ kg^−1^ or greater than 60 mL O_2_ min^−1^ kg^−1^ (as determined post hoc). The Ethics Committee of Manchester Metropolitan University specifically approved this study (Reference Number: 2007.11.04). The investigation was conducted according to the principles of the Declaration of Helsinki and published guidelines [33,34]. Informed consent was obtained from the participants.

**Figure 1:**
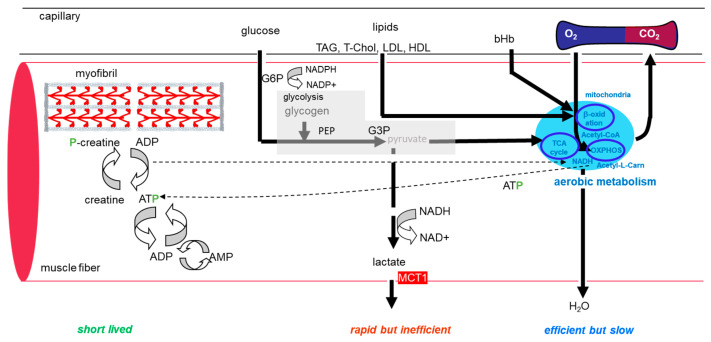
Metabolic processes involved in energy production in muscle. Sketch of the metabolic processes in muscle fibers of interest involving monocarboxylate transporter 1. The position of the studied monocarboxylate transporter 1 (MCT1) is indicated in red. See the list of abbreviations.

Design: Subjects reported to the laboratory on two occasions to estimate aerobic capacity during one session of a two-legged cycling exercise and one session of an exhaustive one-legged cycling exercise in the fasted state in the morning to test the metabolic response. For an overview of the metabolic processes of interest, see Figure 1. The two visits were separated by at least two days. Oxygen uptake, the respiration exchange ratio (RER), and the blood concentrations of selected metabolites, i.e., glucose, triacylglycerol (TAG), total cholesterol (T Chol), low-density lipoprotein and high-density lipoprotein (LDL and HDL), and total ketone (mainly bHb), were monitored in samples collected before and 30 min after exercise, as described previously [35]. Concurrently, biopsies were collected from the musculus vastus lateralis of the nonexercised leg immediately before exercise and 30 min after exercise from the exercising leg. The biopsy material was used to quantify the relative content of muscle metabolites and structural components involved in setting local aerobic capacity, such as the capillary-to-fiber ratio and the percentage of slow-twitch fiber types.

Additionally, cytochrome c oxidase subunit IV isoform 1 (COX4I1) messenger ribonucleic acid (mRNA) was measured as a proxy for mitochondrial volume density. The genotype of rs1049434 was also determined using biopsy material through high-resolution melt polymerase chain reactions. The MOS 36-Item Short-Form Health Survey (SF-36) and an activity questionnaire [36,37] were completed to assess the level of physical activity and current medical health.

Two-legged cycling cardiopulmonary exercise testing: Subjects carried out an incremental test of exercise to exhaustion on a stationary cycle ergometer (Ergometrics Ergoline 800, Jaeger, Bitz, Germany) in an air-conditioned room at 23 °C with the concomitant assessment of cardiopulmonary parameters, essentially as described previously [38]. Age, body mass, and height were measured, and the BMI was calculated during an initial visit. Then, the participants completed a lifestyle questionnaire composed of 31 questions, as modified from a previous short form [37]. Cardiopulmonary measurements were carried out through a mouthpiece with a breath-by-breath technique using a stationary testing device (MetaLyser^®^ 3B, Cortex, Leipzig, Germany). The saddle length was adjusted to a position where the knee was extended at an approximately 175° angle. The subjects were seated with the shoe heel placed on the pedal. The test started with 2 min of baseline recording followed by a 3 min warm-up at 80 W and 80 rpm. The intensity was increased by 25 W every minute until exhaustion. An intensity level was achieved when 80 rpm was held for at least 50 s. Respiration was followed during a cool-down phase of 3 min at 80 W and 80 rpm, followed by 2 min of rest. Test results were recorded at 3 s intervals with the MetaSoft^®^ software (Cortex, Leipzig, Germany) and analyzed offline with the method ‘maximal oxygen uptake’ for absolute and body-mass-related VO_2_max and RER following the exercise. VO_2_max and the corresponding maximum aerobic power output (Pmax) were identified based on the criteria that VO_2_ reached a plateau of a steady maximal value under the imposed high workload when RER was above 1.05 and before VO_2_ dropped, because the pedal rate fell consistently below 70 rpm despite verbal encouragement (for a review, see [38]). The VO_2_ values at the plateau varied within 1% of the average values, and the plateau was maintained on average for over 26 s. The VO_2_max was determined as the highest mean of VO_2_ values averaged over 30 s in the plateau phase. If a VO_2_ plateau did not manifest during the test, the ergospirometry was repeated on a subsequent day.

One-legged cycling exercise: Subjects arrived after an overnight fast and 2 days of reduced physical activity in the laboratory. Under anesthesia, a resting biopsy sample was collected from the vastus lateralis muscle of the non-dominant leg. A 5 mL blood sample was drawn from the cephalic vein into a tube sprayed with dry EDTA (K2E BD Vacutainer^®^, Belliver Industrial Estate, Plymouth, UK) and placed on ice. A 2 mL aliquot was rapidly processed to determine the concentration of selected metabolites. In addition, capillary blood was collected from a finger. Fingertips were cleaned with an ethanol wipe and a small superficial incision was made with the help of a 2.25 mm-depth sterile blood lancet (Safety Lancet; HemoCue^®^; Ängelholm, Sweden). After that, 30 μL of blood was collected from the formed blood droplet with heparinized capillary plastic tubes (Polymer Technology Systems; Indianapolis, IN, USA) to analyze serum metabolites. Subsequently, subjects completed a one-legged exercise test with the dominant leg on the stationary cycle ergometer (Ergometrics Ergoline 800, Jaeger, Bitz, Germany) at a performance-matched intensity and a set cadence of 80 rpm. During the exercise, the pedal for the non-dominant leg was taken off. The saddle length was set to the value used for the two-legged exercise. The shoe of the dominant leg was attached to the pedal with duct tape. The other leg rested on the frame in the middle of the ergometer. Subjects initially performed a warm-up at 15% of the predicted two-legged Pmax, followed by 25 min of exercise at 30% of the two-legged Pmax before the set intensity was ramped up in 10 W increments per minute until exhaustion. A 3 min cool-down phase at 15% of the calculated two-legged Pmax was allowed at the end of the exercise. VO_2_, VCO_2_, and ventilation were monitored with a MetaLyser^®^ 3B system (Cortex, Leipzig, Germany) and VO_2_max and the maximal RER were determined.

Blood serum metabolites: Thirty microliters of capillary blood were used to measure the main metabolic substrates (glucose, triacylglycerol, total cholesterol, LDL and HDL, and/or total ketones) using a portable whole blood test system (CardioCheck ST Analyzer, Polymer Technology Systems; Indianapolis, IN, USA). The glucose concentration was measured in the first minute after collection. LDL and VLDL cholesterol serum concentrations were calculated as previously described [39]. The coefficient of variation for repeated measurements was 2.6% for glucose and 3.9% for triglycerides. 

Biopsy: An experienced physician collected muscle samples (30–100 mg) taken from the vastus lateralis muscle at the point of maximal thickness. The overlying skin was shaved and sterilized (Videne Antiseptic Solution, Ecolab, Saint Paul, MN, USA). A sterile drape from a wound care pack (Premier, Shermond Bunzel Retail & Health Care Supplies Limited, Enfiled, Middlesex, UK) ensured sterile conditions. For local anesthesia, 1 mL of 2% Lidocaine was injected subcutaneously. Within 5 min, a 5 mm incision was made with a scalpel and a muscle sample was extracted and immediately processed by a skilled investigator. Before exercise, the samples were collected with a rongeur using the conchotome technique, while post-exercise samples were collected using a biopsy needle (TSK Acecut 11G, Emergo Europe, The Hague, The Netherlands). Firm pressure was applied to the biopsy site until the bleeding stopped. The wound was then closed (Steri-Strip, 3M Health Care, Germany) and dressed (Mepore Ultra, Molnlycke Healthcare, Sweden). Subjects were discharged with a pressure bandage for the first 4 h after the biopsy sample to reduce further bleeding. Biopsy samples were rapidly frozen in liquid nitrogen while shaking the sample. Pre-exercise samples were cut into two pieces before being frozen, one being mounted with Tissue-Tek^®^ O.C.T. TM Compound (Weckert Labortechnik, Kitzingen, Germany) on cork for histological analysis before freezing. The samples were stored airtight in a 2 mL tube (Eppendorf) until further processing.

Muscle structure: The capillary-to-fiber ratio and fiber type distribution were determined with quantitative microscopy, as previously described [6]. In brief, capillaries were detected in 14 μm-thick cryosections prepared from pre-exercise biopsies with a lectin antibody. The capillary-to-fiber ratio was quantified in the sections using an established microscopic procedure based on recordings with an Axioskop 2 system (Carl Zeiss, Oberkochen, Germany) and subsequent quantification using the ImageJ software (version 1.6.0_33; http://imagej.nih.gov/ij). 

Additionally, the mean cross-sectional area (MCSA) and percentage of slow (type I) and fast (type II) muscle fibers were determined on muscle cross-sections stained with myosin isoform-specific antibodies, as previously described [38]. Subsequently, the area percentage of slow-type fibers was calculated. The values of at least 24 representative fibers per subject were analyzed. 

Muscle metabolites: Polar and non-polar metabolites, i.e., lipids, in skeletal muscle were analyzed with mass spectrometry, essentially as described previously [35]. In brief, extracts were prepared from ~5 mg of muscle sample in a corresponding volume of cold methanol:methyl-tert-butyl ether:H_2_0 = 360:1200:348 using a full glass Potter-type homogenizer. The polar phase was recovered, dried down under a stream of nitrogen, and reconstituted in 100 μL of 50 mM ammonium acetate in acetonitrile:water 9:1 (*v*/*v*) using high-quality high-performance liquid chromatography grade (Chromasolv, Sigma-Aldrich, Buchs, Switzerland).

Subsequently, 1 μL of sample from the polar phase was injected into a nanoAquity UPLC (ultra-high-performance liquid chromatography; Waters, Baden-Dättwil, Switzerland) to first separate metabolites across a BEH-Amide capillary column (200 μm × 150 mm, 1.7 μm particle size; Waters, Baden-Dättwil, Switzerland) using a gradient of ammonium acetate in acetonitrile and water. Fractions were automatically injected into the coupled Q Exactive™ Hybrid Quadrupole-Orbitrap Mass Spectrometer (Thermo Fisher Scientific, Reinach, Switzerland) via a nano-electrospray ionization source, and mass spectroscopy (MS) data were acquired using negative polarization. Ion fragmentation was performed over a mass range of 80 to 1200 *m*/*z* at a resolution of 70,000 (MS) and 25,000 (MSMS). For the detection of lactate, alternative settings were applied with positive electrospray ionization over a mass range of 50 to 1500 Daltons.

Lipids were characterized in extracts being prepared from grinding ~15 mg of muscle sample with the help of a ball mill into cold chloroform:methanol = 1:1. The non-polar phase was flash-frozen in liquid nitrogen and samples were directly infused into a Thermo LTQ Orbitrap Mass Spectrometer (Thermo Fisher) that was operated in negative electrospray ionization mode. The MS data were acquired with settings for independent fragmentation of all ions and stepped collision energy. 

Datasets were processed with Progenesis QI (Nonlinear Dynamics, A Waters Company), followed by peak picking on an aggregated ion intensity map to detect and quantify the compound ions of interest based on the consensus retention times of observed mass-to-charge ratio (*m*/*z*) signals for the major ions in all the samples. The detected compound ions were further annotated through database searches, including the Human Metabolome Database (HMDB, www.hmdb.ca), to match observed fragmentation spectra with theoretical fragmentation spectra within a tolerance of 50 milli Daltons. Lipid compounds were identified by comparison to the lipidmaps Database (lipidmaps.org) for a match in the neutral mass with a tolerance of 60 milli Daltons. 

Quality controls were run on individual and mixed samples to determine technical accuracy based on 20 selected compounds (amino acids, nucleotides, and metabolic intermediates) in mixed samples using Quan Browser (Xcalibur, Thermo Fisher Scientific) and 61 further abundant ions using Progenesis QI software (Nonlinear Dynamics). The coefficients of variation for biological and technical replicas demonstrated values near or below 20%. 

Then, 14 polar metabolites of interest, associated with aerobic metabolism (Figure 1), and 256 lipids were detected by UPLC-MS and quantified. These included acetyl-co-enzyme A, acetyl-L-carnitine, adenosine triphosphate, adenosine diphosphate, adenosine monophosphate, β hydroxy butyrate, creatine, flavin adenine dinucleotide, glycerol-3-phosphate, nicotinamide adenine dinucleotide, nicotinamide adenine dinucleotide phosphate, phosphoenolpyruvic acid, and phosphocreatine (Table S1). In addition, the simplest structure of the polymeric glycogen, i.e., α-D-Glucopyranosyl-(1->4)-[α-D-glucopyranosyl-(1->6)]-α-D-glucopyranosyl-(1->4)-α-D-glucopyranose, was analyzed (https://hmdb.ca/metabolites/HMDB0000757). The data were normalized to the average, or median in the case of lipids, of values for all samples before and after exercise for further statistical analyses.

In addition, glycogen concentration in muscle biopsies was quantified per total muscle protein, as described [38]. In brief, cryosections, corresponding to a volume of 1 mm^3^ tissue, were homogenized in 100 μL of phosphate-buffered saline, including an inhibitor cocktail (1 mL PBS + 9 mL dH_2_O + 1 complete Mini, EDTA-free tablet (Sigma Aldrich, Buchs, Switzerland)). Subsequently, total protein content and glycogen content were measured in homogenates with the Pierce BCA Protein Assay Kit (Thermo Scientific, Waltham, MA, USA) and the Assay Kit (Abcam, Cambridge, UK), respectively, according to standard instructions. 

Genotyping: Genomic DNA was extracted from homogenates of muscle biopsies in RIPA buffer (10 mm^3^ per 200 μL). The samples were diluted 1:20 in pure water and subjected to specific polymerase chain reactions with defined reagents on a magnetic induction cycler (Mic Real-Time Polymerase Chain Reactions (PCR) System, Bio Molecular Systems; Labgene, Châtel-St-Denis, Switzerland). The specific conditions included the use of 200 nM sense (AGATGTTGCTGGGAAGCCAA) and antisense oligonucleotides (CTTCAGCCCCATGGATTCAG), in combination with a SensiFAST HRM Mix (Meridian Biosciences; BIO32005, Labgene, Châtel-St-Denis, Switzerland). The temperature protocol consisted of 40 cycles of denaturing for 15 s at 95 °C, annealing for 15 s at 55 °C, and extension for 30 s at 72 °C before high-resolution melting curve analysis was conducted with a gradient from 50 to 95 °C with increments of 0.1 °C s^−1^. Samples were run against genotype references established in previous PCR experiments based on Sanger sequencing of the amplicons by a commercial provider (Microsynth, Balgach, Switzerland).

COX4I1 mRNA: The transcript levels of COX4I1 and glyceraldehyde-3-phosphate dehydrogenase (GAPDH) were quantified as described previously [40]. In brief, total RNA was isolated from 5 mm^3^ of biopsy sample using the RNeasy Mini Kit (Qiagen, Hombrechtikon, Switzerland) and 600 ng was reverse-transcribed using the Omniscript RT Kit (Qiagen, Hombrechtikon, Switzerland) with random hexamers (Qiagen, Hombrechtikon, Switzerland). cDNAs were amplified from 6 ng of cDNA and quantified via polymerase chain reactions. The primers used were as follows: For COX4I1 (forward, 5′-GCC ATG TTC TTC ATC GGT TTC-3′; reverse, 5′-GGC CGT ACA CAT AGT GCT TCT G-3′), and for GAPDH (forward: 5′-GGA GCG AGA CCC CAC TAA CA-3′; reverse: 5′-GCC TTC TCC ATG GTG GTG AA-3′). Reactions were run at a primer concentration of 100 nM using KAPA SYBR Fast Universal Master mix (Applied Biosystems, KK4603, Merck & Cie, 9470 Buchs, Switzerland) and run on a StepOne™ Real-Time PCR System (Applied Biosystems, Fisher Scientific, Reinach, Switzerland). The thermal protocol consisted of 40 cycles of denaturation for 15 s at 95 °C, annealing, and extension at 60 °C. The specificity of amplification was assessed based on melting curves versus a reference that was constituted from a dilution of a PCR sample, for which specificity was confirmed by commercial microsequencing (Microsynth, Balgach, Switzerland). Relative transcript amounts were calculated using the comparative CT method, taking the amplification efficiency for each template into account using the comparative method for the threshold cycle for target amplification, as described [41]. For each sample, transcript signals were standardized to the estimated GAPDH transcript levels.

Statistics: Statistical effects were assessed with analysis of variance for repeated measures for the repeated factor of exercise and the factor rs1049434 genotype for a dominant effect model of the T-allele, i.e., TT/AT vs. AA genotypes, and effects were localized with the post tests of Tukey or Holm (JASP V0.18.3, University of Amsterdam, https://jasp-stats.org/download/). Statistical analysis of exercise and genotype effects on lipid compounds was carried out with a permutation-based statistical analysis of microarrays (SAM) that calculates false-discovery rate-adjusted probabilities for differences between groups based on a q-value, as described [38]. The level of significance was set to a 5% threshold. Data are displayed individually in raincloud plots or box plots with the following designations (line: median; cross: mean; box: data from 1st to 3rd quartile; whiskers: ±1.5 × interquartile range). Linear relationships were assessed with Pearson’s moment correlations and considered present if |r| ≥ 0.5 and *p* < 5%.

## 3. Results

### 3.1. Characteristics of Aerobic Capacity

The subjects demonstrated average values for healthy males (peak aerobic power output: 313.8 ± 54.2 W; maximal oxygen uptake: 4089.4 ± 707.4 L O_2_ min^−1^) and conducted a one-legged ramp exercise under a maximal output of 193.3 ± 41.9 W (Table 1). 

### 3.2. Effects of Exercise on Metabolic Substrates in Blood

**Figure 2:**
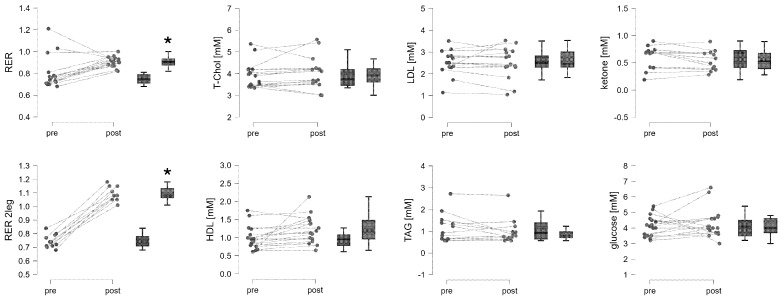
One-legged exercise induces changes in blood gases and metabolites. Spaghetti and box plots of blood metabolite and blood gas values before and 30 min after exhaustive one-legged ramp exercise (n = 22). RER 2-leg corresponds to the respiration exchange ratio during two-legged cardiopulmonary exercise testing. *, *p* < 0.05 vs. pre-test, repeated-measures ANOVA with post hoc Tukey/Holm test.

After one-legged exercise, the respiration exchange rate (RER) increased by 13.1%, the blood HDL concentration increased by 28.5%, and the blood triacyl-glyceride concentration decreased by 21.7%. In contrast, other substrate metabolites in capillary blood were unaffected (Figure 2).

### 3.3. Exercise-Induced Effects on Muscle Metabolites

**Figure 3:**
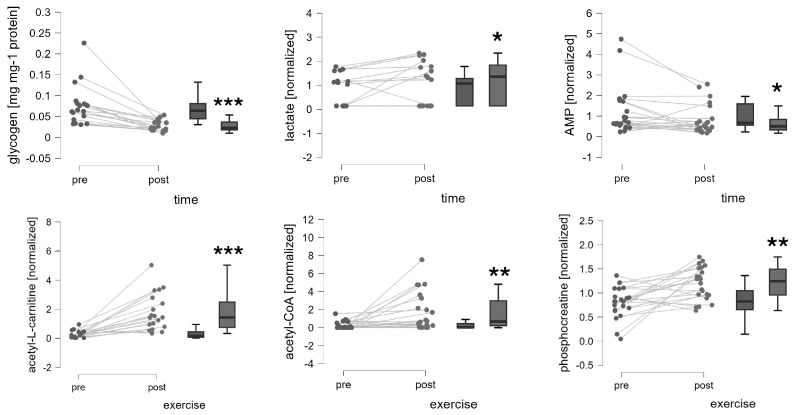
Metabolic changes in the knee extensor muscle are induced by one-legged exercise to exhaustion. Spaghetti and box plots of the values for muscle metabolites for the 22 assessed subjects pre- vs. post-exercise in the exercised leg. *, **, and *** denote *p* < 0.05, <0.01, and <0.001, respectively. Repeated ANOVA with post hoc test of Tukey/Holm.

Greater alterations were observed in the vastus lateralis muscle 30 min post-exercise for prominent metabolites that affect flux through the Krebs cycle, i.e., glycogen (−58.6%), simple glycogen (HMDB0000757; −63.3%), lactate (+41.7%), acetyl-CoA (+448.1%), and acetyl-L-carnitine (+548.0%; Figure 3), which are in line with recent reports [42]. The concentration of AMP (−32%) decreased, and the phosphocreatine concentration (+48%) increased 30 min after exercise. The lactate concentration increased post-exercise (+13%) but was not statistically significant. The concentration of bHb in muscle was not affected by exercise (*p* = 0.454).

The 256 detected lipidic compounds in vastus lateralis muscle demonstrated an overall 25.6% increase 30 min post-exercise (*p* < 0.001) and a trend for association with the *MCT1* genotype (*p* = 0.084). No single lipid demonstrated an exercise-induced alteration. 

### 3.4. MCT1 Genotype and Physiological Variables

Variability in the percentage and area percentage of slow-type muscle fibers, but not any other physiological parameters related to metabolic and contractile performance, was associated with the rs1049434 genotype (Table 1). Compared to carriers, non-carriers of the T-allele demonstrated a 37% greater area percentage and a 25% greater percentage of slow-type fibers than did T-allele carriers.

### 3.5. Differences in Metabolic Concentrations Associated with the MCT1 Genotype

**Figure 4:**
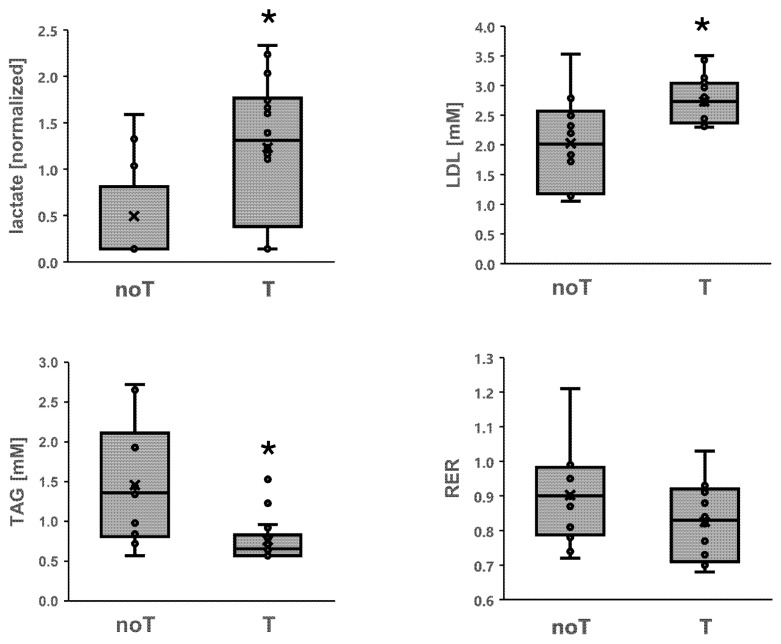
Metabolites demonstrating genotype differences, irrespective of exercise. Box plots for muscle lactate concentration in M. vastus lateralis, LDL, and TAG in blood, as they demonstrate different levels in T-allele carriers (n = 15) than in non-carriers of rs1049434 (n = 7), irrespective of exercise. * *p* < 0.05 vs. AA. ANOVA with post hoc test of Tukey/Holm. RER: *p* = 0.059.

The concentrations of lactate in the knee extensor muscle (*p* = 0.049) and blood concentrations of LDL (*p* = 0.015) and TAG (*p* = 0.001) demonstrated rs1049434 genotype-associated variability irrespective of exercise, with T-allele carriers reaching 150.1% higher muscle lactate concentrations, 45.9% higher blood LDL concentrations than non-carriers, and 53.0% lower blood TAG concentrations than non-carriers of the T-allele (Figure 4). The RER trended toward a rs1049434 genotype difference (T vs. no T-allele: 0.93 vs. 0.90, *p* = 0.059).

### 3.6. Interaction Effects between MCT1 Genotype and Exercise on Metabolite Concentrations

**Figure 5:**
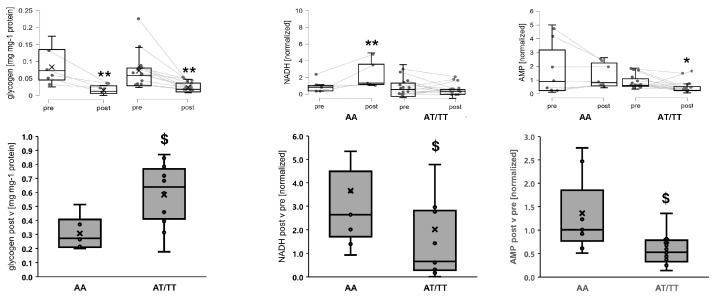
Muscle metabolites demonstrating *MCT1* genotype differences in exercise-induced changes. Combined spaghetti and box plots for the concentrations of glycogen, NADH, and AMP in M. vastus lateralis, and LDL and TAG in blood, which demonstrated an interaction between exercise and rs1049434, i.e., the studied 15 carriers and 7 non-carriers of the T-allele. * and ** denote *p* < 0.05 and <0.01 vs. pre-test for the respective situation. $ denotes *p* < 0.05 vs. AA. ANOVA with post hoc test of Tukey/Holm.

Muscle levels of glycogen, NADH, and AMP were affected in an *MCT1* genotype-dependent manner by exercise (Table 2; Figure 5). Glycogen concentration was 63% more reduced in carriers than non-carriers of the T-allele. NADH was selectively increased in AA genotypes 30 min post-exercise (+314%) but not affected in AT/TT genotypes (*p* = 1.0). AMP concentration was selectively reduced in T-allele carriers post-exercise.

Fourteen lipidic compounds in vastus lateralis muscle demonstrated *MCT1* genotype-associated differences in exercise-induced level alterations (Table S2). This concerned a long-chain triglyceride (TG 78:5; O3), three long-chain co-enzyme As (CoA 18:0; O4; CoA 18:3; O4; CoA 20:3; O3), as well as four glycerophospholipids, all of which were selectively increased 30 min after exercise in non-carriers of the T-allele when being unaffected T-allele non-carriers.

### 3.7. Correspondence of MCT1 Genotype-Associated Structural and Metabolic Parameters

Several correlations were identified for the assessed metabolic factors and markers of the slow and/or oxidative muscle phenotypes (Table S3). The relationships between the percentage of slow fibers and the concentration of TAG in the blood prior to and after exercise were r = 0.58 and r = 0.61, respectively. The fold changes in bHb in capillary blood were correlated with capillary density (r = −0.67) and LDL (r = −0.57). Furthermore, the muscle glycogen concentration after exercise (r = 0.79) and the LDL concentration in the blood before (r = −0.65) and after exercise (r = −0.56) were correlated with the mitochondrial marker, GAPDH, which is related to the concentration of COX4I1 mRNA.

## 4. Discussion

This study aimed to analyze whether perturbations in working muscle related to intramuscular lactate and ketone metabolism during exhaustive exercise result from the nonsynonymous gene polymorphism rs1049434 for monocarboxylate transporter 1 (*MCT1*). This transporter affects the trans-capillary transport rates of the aforementioned compounds [8]. To the best of our knowledge, the extent to which rs1049434 impacts the concentration of ketone bodies, the intramuscular concentration of lactate, and the inter-related reactant NADH [43], as well as dependent metabolic TCA and glycolytic pathways (reviewed in [44]), is not known. However, rs1049434 affects the variability of blood lactate accumulation with exhaustive exercise via effects on (trans-) capillary lactate efflux from the muscle [8] and possibly influx into slow-type-muscle fibers (reviewed in [20]). Furthermore, evidence exists in animal and human models for increased MCT1 expression with physical training [45,46]. Here, we detected that non-carriers of the T-allele of rs1049434 (that is, AA genotypes) demonstrated a greater reduction in glycogen concentration and an increase in NADH concentration than did T-allele carriers (AT/TT genotypes) after comparing rates of respiration. This suggests a reduced consumption of NADH in non-T-carriers for converting pyruvate to lactate. Concomitantly, the intramuscular concentration of lactate was greater, independent of exercise, in the T-allele carriers. Furthermore, rs1049434- and exercise-dependent differences were noted for the AMP concentration, whose charging is mainly related to the TCA cycle’s combined action and auxiliary adenylate kinases. The present novel observations support the view that the studied nonsynonymous genetic influence of *MCT1* shifts the contribution of glycolytic metabolism to the aerobic production of energy for muscle work.

Intriguingly, exercise only marginally insignificantly affected the muscle lactate concentration (*p* = 0.082). This observation may be related to the timing of the sample point to 30 min after exercise when metabolic perturbations possibly decrease to the considerable efflux rates of lactate into the bloodstream [47], and muscle levels of lactate have rarely been reported [48]. Our observations do not support the null hypothesis of an absent upregulation of lactate concentration by the deployed type of exhaustive one-leg exercise. 

The degree to which exercise-induced changes in the muscle lactate concentration immediately after exhaustion may occur early in a rs1049434-dependent manner remains to be tested. Similarly, we found that the variability in the concentrations of bHb in muscle (*p* = 0.454) and capillary blood (*p* = 0.203) was not associated with exercise or rs1049434 (*p* = 0.308 and 0.510, respectively). These findings strongly indicate that in the present study, ketone metabolism was affected neither by exercise nor rs1049434. In our study, the ketone concentration in capillary blood was slightly above normal after an overnight fast. Therefore, the extent of this nutritional intervention and the relatively short duration of exercise, which did not affect bHb concentrations in blood and muscle and prevented addressing possible effects of the *MCT1* genotype on endurance or performance and overall ketone metabolism, is unknown.

For rejecting or accepting the null hypothesis of rs1049434-associated variability in metabolites, the consideration of identified linear relationships between hallmarks of the muscle phenotype and metabolite concentration is of interest (Table S3). First, for rs1049434- and exercise-dependent genes, metabolite correspondences were identified with structural hallmarks whose variability was also associated with rs1049434. First, the glycogen concentration correlated positively (r = 0.59) with the percentage of slow-type fibers before exercise. We interpreted this to suggest that recruited slow fiber types mainly contribute to the observed exercise-induced activation of glycolysis. Second, the genotype-dependent and exercise-dependent concentration of the TAG substrate for muscle metabolism in capillary blood had a linear relationship with the slow type of muscle area (r = 0.58 and 0.61). This finding provides novel information that the studied *MCT1* genotype affects the variability in blood-borne levels of lipidic substrates in connection with the composition of slow-type muscle fibers. Intriguingly, the fold changes in TAG (r = −0.53) and muscle glycogen (r = 0.79) after exercise were correlated with the mRNA levels of the mitochondrial marker COX4I1/GAPDH.

Interestingly, the TAG concentration, which was three times greater in AA genotype carriers than in T-allele carriers, also demonstrated a trend toward a genotype x exercise interaction (*p* = 0.084). Indeed, upon closer inspection of genotype differences based on an additive model, we noted a trend (*p* = 0.074) for an influence of the rs1049434 genotype on the RER during one-legged exercise, which was lower in the AT genotypes before exercise (0.71) than in the AA (0.88) and TT (0.85) genotypes. These observations motivate further investigations into the role of MCT1 in selecting substrates during exhaustive types of endurance exercise, the involved mechanisms of which likely include metabolic organs, such as the liver and the muscle compartment [49,50]. In contrast, the fold changes in the bHb concentration in capillary blood correlated with capillary density (r = −0.67) and the capillary-to-fiber ratio (r = −0.60), neither of which factors affected the rs1049434 genotype (Table 2). This indicates a relevant quantitative influence of the structural aspects of trans-capillary import and export for this ketone body rather than the MCT1 channel itself. In addition, the muscle lactate concentration did not correlate with any of the studied hallmarks of the slow oxidative muscle phenotype or indices of muscle perfusion, i.e., capillary density or the capillary-to-fiber ratio (Table S3). Considering the association between variability in muscle lactate concentration and rs1049434, this finding suggests a causal relationship between variability and decoupled MCT1 transporter activity.

Intriguingly, we observed an interaction effect of rs1049434 and exercise on the muscle concentration of AMP when the ADP and ATP concentrations were unaffected (Figure 5; Table 2). Similarly, phosphocreatine tended to have an effect of exercise × rs1049434 (*p* = 0.078), and a selective increase in phosphocreatine was noted in the T-allele carriers (+69%, *p* = 0.003) when no such effect was observed in the non-carriers (*p* = 0.537). The effect of genotype x exercise on AMP concentration localized to a selective reduction in T-allele carriers post-exercise. AMP typically arises from the action of adenylate kinase on two ADP molecules to produce one molecule of the main energy carrier ATP that recharges creatine to produce phosphocreatine via the action of creatine kinase [51]. AMP may also deaminate into inosine monophosphate and ammonia, and this is enhanced during exercise when the capacity to re-phosphorylate ADP is impaired [52]. The deployed mass spectrometric analysis was not geared toward detecting inosine monophosphate. Our novel observation of lower AMP levels in T-allele carriers, with a seemingly lower potential for NADH-driven ATP synthesis in mitochondria post-exercise, is suggestive of a compensatory reaction involving myokinase or AMP deaminase in an attempt to maintain high concentrations of high-energy phosphates in T-allele carriers of rs1049434.

Participants of the one-legged cycling exercise test did not show differences in performance-related parameters, Pmax and VO_2_max, or body mass. The identified genotype-associated differences in lactate, NADH, AMP, and glycogen after exhaustive one-legged ramp exercise reflected considerable differences in muscle metabolism. Owing to genotype-associated differences in blood LDL and TAG, we expect that longer durations of exercise below the anaerobic threshold are affected by gene polymorphisms rs1049434. This activity importantly relies on the aerobic combustion of lipids [13]. This notion is corroborated by the observation that the levels of three long-chain acyl co-enzyme As and a triglyceride (Table S2) were selectively increased in AA genotypes 30 min after exercise. These alterations, as they relate to lipid hydrolysis and synthesis and reflect intramuscular stores of lipids (reviewed in [35]), suggest an enhanced drive for the handling of intramuscular lipid stores in the non-carriers compared to the carriers of the T-allele during and immediately after exercise.

These findings suggest that non-carriers of the T allele are more likely to be endurance-oriented athletes, whereas the AT/TT genotype is associated with elite sprint/power athletic status [23,26]. This contention is in line with our observation that T-allele carriers demonstrated a greater anaerobic glycolytic strain based on higher muscle lactate levels and is intuitive for athletes competing, for instance, in 400 m runs, where the highest blood lactate concentrations are detected. In contrast, as AA genotypes have a stronger preference for the metabolic pathway via glycogen, in combination with a slower muscle fiber genotype, there is likely a comparative advantage for endurance exercise. However, performance differences with a standard laboratory test have not been presented to arise due to the *MCT1* gene polymorphism rs1049434 [25]. In this regard, it should also be noted that some papers used antisense annotation of the polymorphism investigated in this study [22]. Thus, the observed shift to increased consumption of glycogen and increased NADH, which drives ATP production in AA genotypes, and the effects of rs1049434 on the metabolic substrates TAG and LDL suggest that future studies should address the effect of MCT1 on the metabolic economy of exercise. In this respect, our investigations also suggested that inter-individual differences in lactate levels should be interpreted in relation to the genotype investigated here rather than the state of local aerobic fitness. This might influence training recommendations and coaches that measure blood lactate levels for training intensity prescription on a group level.

Our pilot investigation has several limitations. First, due to organizational and ethical issues, we addressed the metabolic effects of exercise on the knee extensor muscle for 30 min and not during exercise or immediately after exhaustion. Consequently, we may have missed some effects (such as potentially affecting lactate concentrations in muscle, which here were only presented as a trend, *p* = 0.080). In contrast, others (such as increased phosphocreatine levels post-exercise) were not expected. We also note that we did not assess lactate concentrations in capillary blood due to the failure of the selected equipment to detect this compound and the unavailability of other equipment. Finally, we assessed metabolites in muscle tissue extracts to determine the effective contribution of the quantitatively most-abundant muscle fibers. Whether different cell types influence the observed metabolite levels remains to be elucidated. 

## 5. Conclusions

Differences in metabolic compounds in muscle and capillary blood revealed the anticipated metabolic reactions in lactate metabolism between carriers and non-carriers of the T-allele of rs1049434. The observed differences aligned with the suspected increased accumulation of lactate and reduced NADH concentration in T-allele carriers, which prevailed 30 min after exercise for T-allele carriers. 

Moreover, our investigation did not identify the suspected genetic influence on the transport of ketone metabolism due to the relatively short impact of exercise on subjects after an overnight fast. Genotype and exercise interaction effects on ‘empty’ high-energy phosphate, AMP, and phosphocreatine suggested that compensatory reactions involving myokinase, adenylate kinase, or AMP deaminase are indicated for future studies. Importantly, we identified an association between rs1049434 and variability in the studied knee extensor muscle composition in terms of slow-type fibers, which are quantitatively related to exercise-affected metabolic substrates, foremost glycogen, and TAG. In turn, post-exercise concentrations of the latter also correlated with the mitochondrial COX4I1/GAPDH mRNA ratio. Conversely, the concentrations of a few metabolites not associated with the studied *MCT1* genotype, i.e., bHb and T-Chol, corresponded to perfusion indices (i.e., capillarization). This highlighted that the studied nonsynonymous *MCT1* gene polymorphism may only partially overcome the structural limitations on the trans-capillary import of substrates for aerobic muscle metabolism during exercise. The *MCT1* gene polymorphism appeared to play a significant role in selecting glycogen and triacylglycerol (TAG) for muscle metabolism during exercise, although this did not result in major differences in respiration during the type of exercise performed.

## Figures and Tables

**Table 1:** Biometric characteristics of the studied healthy male subjects. Mean ± SD. Abbreviations: BMI, body mass index; Bpsys, systolic blood pressure; Bpdia, diastolic blood pressure; COX4I1/GAPDH, ratio between the levels of messenger ribonucleic acids for cytochrome c oxidase subunit IV isoform 1 and glyceraldehyde-3-phosphate dehydrogenase; MCSA, mean cross-sectional area; mRNA, messenger ribonucleic acid; Pmax 1leg, maximum aerobic power output during one-legged exercise to exhaustion; Pmax 2leg, maximum aerobic power output during two-legged exercise to exhaustion; VO_2_max 1leg, maximal oxygen uptake during one-legged exercise to exhaustion; VO_2_max 2leg, maximum maximal oxygen uptake during two-legged exercise to exhaustion. Bold underlined *p*-values are considered to be relevant. N = 15 for AT/TT (10/5), and n = 7 for AA.

	**Age**	**Body mass**	**Height**	**BMI**
	**(years)**	**(kg)**	**(m)**	**(kg m^−2^)**
AA	29.3 ± 3.1	79.0 ± 5.3	1.82 ± 0.07	24.7 ± 1.5
AT/TT	26.5 ± 1.5	78.3 ± 2.3	1.80 ± 0.07	23.7 ± 0.7
p	0.365	0.888	0.263	0.467
η^2^	0.043	0.001	0.065	0.028
	**Pmax 2leg**	**Pmax 1leg**	**VO_2_max 2leg**	**VO_2_max 1leg**
	**(Watt)**	**(Watt)**	**(mL O_2_ min^−1^)**	**(mL O_2_ min^−1^)**
AA	328.6 ± 19.2	200.8 ± 21.8	4254.4 ± 247.2	3538.983 ± 354.1
AT/TT	307.0 ± 14.7	183.7 ± 9.3	3935.8 ± 181.4	3320.607 ± 137.3
p	0.403	0.400	0.324	0.487
η^2^	0.035	0.038	0.049	0.026
	**VO_2_max**	**Bpsys**	**Bpdia**	
	**(mL O_2_ min^−1^ kg^−1^)**	**(mm Hg)**	**(mm Hg)**	
AA	54.5 ± 3.0	121.5 ± 1.6	70.8 ± 2.5	
AT/TT	51.3 ± 1.8	124.1 ± 3.2T	74.4 ± 2.2	
p	0.342	0.662	0.398	
η^2^	0.045	0.014	0.052	
	**MCSA**	**Slow-fiber area**	**Slow-type fiber**	
	**(µm^2^)**	**(%)**	**(%)**	
AA	7871.0 ± 715.2	44.2 ± 3.1	47.8 ± 4.1	
AT/TT	6339.4 ± 468.5	32.2 ± 2.6	38.2 ± 1.9	
p	0.105	**0.021**	**0.034**	
η^2^	0.147	0.292	0.283	
	**COX4I1/GAPDH**	**Capillary density**	**Capillary-to-fiber ratio**	
	**mRNA**	**(mm^−2^)**		
AA	51.6 ± 166.2	296.9 ± 14.7	2.78 ± 0.38	
AT/TT	73.5 ± 10,664.8	289.8 ± 11.1	2.12 ± 0.15	
	−98%	2%	31%	
p	0.437	0.714	0.063	
η^2^	0.03	0.007	0.162	

**Table 2:** Main effects of exercise and the *MCT1* genotype on muscle metabolites. List of effect sizes and *p*-values for the interaction effects between exercise (pre and post) and genotype (genotypes of rs1049434) gene polymorphisms. Repeated ANOVA for factors exercise x genotype for the dominant (AA vs. AT/TT) model for the effects of gene polymorphism rs1049434. Bold underlined *p*-values are considered to be relevant and were followed up with post hoc tests. Abbreviations: Acetyl-CoA, acetyl-co-enzyme A; ADP, adenosine diphosphate; ALCAR, L-acetylcarnitine; AMP, adenosine monophosphate; ATP, adenosine triphosphate; bHb, 3-hydroxybutyric acid (β-hydroxybutyrate); FAD, flavin adenine dinucleotide; HDL, high-density lipoprotein; LDL, low-density lipoprotein; NADH, reduced form of nicotinamide adenine dinucleotide; NADP, nicotinamide adenine dinucleotide phosphate; RER, respiration exchange ratio during one-legged exercise to exhaustion; RER 2leg, respiration exchange ratio during two-legged exercise to exhaustion; T-Chol, total cholesterol; TAG, triacylglyceride.

		Dominant Model		
		Exercise		Exercise x
			Genotype	Genotype
***blood gas***				
RER 1leg	p	**0.008**	0.761	0.392
	η^2^	0.2	0.003	0.016
RER 2leg	p	**<0.001**	0.442	0.196
	η^2^	0.908	0.004	0.006
***capillary blood***				
glucose	p	0.435	0.655	0.418
	η^2^	0.017	0.009	0.018
bHb	p	0.203	0.308	0.887
	η^2^	0.017	0.092	<0.001
T-Chol	p	0.664	0.574	0.787
	η^2^	0.002	0.075	0.006
LDL	p	0.9	**0.015**	0.732
	η^2^	<0.001	0.32	<0.001
HDL	p	**0.033**	0.646	0.199
	η^2^	0.101	0.01	0.033
TAG	p	0.069	**0.001**	**0.084**
	η^2^	0.022	0.53	0.019
***in muscle***				
bHb	p	0.454	0.51	0.518
	η^2^	0.017	0.013	0.012
glucose-6-phosphate	p	0.56	0.081	0.65
	η^2^	0.009	0.083	0.005
glycogen	p	**<0.001**	0.561	**0.027**
	η^2^	0.371	0.012	0.053
phosphoenol pyruvate	p	0.384	0.145	0.514
	η^2^	0.016	0.058	0.009
lactate	p	0.082	**0.049**	0.991
	η^2^	0.043	0.196	<0.001
glycerol-3-phosphate	p	0.944	0.586	0.582
	η^2^	<0.001	0.009	0.006
Acetyl-CoA	p	**0.005**	0.833	0.643
	η^2^	0.21	0.001	0.004
ALCAR	p	**<0.001**	0.835	0.754
	η^2^	0.415	<0.001	0.001
FAD	p	0.193	0.126	0.653
	η^2^	0.038	0.062	0.004
NADH	p	0.078	0.064	**0.015**
	η^2^	0.05	0.114	0.103
NADP	p	0.199	0.269	0.065
	η^2^	0.05	0.212	0.136
AMP	p	**0.039**	0.799	**0.028**
	η^2^	0.036	<0.001	0.178
ADP	p	0.991	0.402	0.823
	η^2^	<0.001	0.009	0.002
ATP	p	0.831	0.982	0.987
	η^2^	<0.001	<0.001	<0.001
creatine	p	0.414	0.339	0.631
	η^2^	0.008	0.035	0.003
phosphocreatine	p	**0.009**	0.961	**0.078**
	η^2^	0.167	<0.001	0.069

## Data Availability

The original contributions presented in the study are included in the article/Supplementary Materials, further inquiries can be directed to the corresponding author.

## Supplementary Materials

The following supporting information can be downloaded at: https://www.mdpi.com/article/10.3390/genes15070918/s1. Table S1: Characteristics of the detected metabolic compounds. Table S2: Muscle lipids that demonstrated rs1049434 genotype-associated differences in the exercise response. Table S3: Structure–function relationships.
