# Supplementary material for: T-Allele Carriers of Mono Carboxylate Transporter One Gene Polymorphism rs1049434 Demonstrate Altered Substrate Metabolization during Exhaustive Exercise
Source: Genes (Basel). 2024 Jul 14;15(7):918. doi: 10.3390/genes15070918 (PMC11275951; doi:10.3390/genes15070918)
Supplement: Supplementary file 1 [file genes-15-00918-s001.zip › Table S1 R2.pdf]

Table S1: Characteristics of the detected metabolic compounds in vastus lateralis muscle. Abbreviations: HMDB, identifier according to the human data base (https://hmdb.ca), UPLC-MS ESI, ultrahigh performance liquid chromatography-tandem mass spectrometry with electron spray ionization in negative mode; UPLC-MS ESI+, ultrahigh performance liquid chromatography-tandem mass spectrometry with electron spray ionization in positive mode.

compound	***description***	***Compound ID***	***m/z***	***method***
bHb	3-hydroxybutyric acid	HMDB00357	103.0387645	UPLC-MS ESI-
Ac-CoA	acetyl-CoA	HMDB01206	403.556154	UPLC-MS ESI-
ALCAR	L-acetylcarnitine	HMDB00201	202.1101379	UPLC-MS ESI-
ADP	ADP	HMDB00061	426.0270973	UPLC-MS ESI-
AMP	adenosine monophosphate	HMDB00045	346.058787	UPLC-MS ESI-
ATP	adenosine triphosphate	HMDB00538	505.9930942	UPLC-MS ESI-
Creatine	creatine	HMDB00064	261.1325515	UPLC-MS ESI-
FAD	flavin adenine dinucleotide	HMDB01248	784.1531751	UPLC-MS ESI-
G3P	glycerol_3_phosphate	HMDB00126	171.005828	UPLC-MS ESI-
G6P	glucose-6-phosphate	HMDB01401	259.023177	UPLC-MS ESI-
simple glycogen	α-D-Glucopyranosyl-(1->4)-[α-D-glucopyranosyl-(1->6)] -α-D-glucopyranosyl-(1->4)-α-D-glucopyranose	HMDB0000757	666.2264	UPLC-MS ESI-
Lactate	lactate	HMDB00190	174.086792	UPLC-MS ESI+
NADH	nicotinamide adenine dinucleotide	HMDB01487	664.120719	UPLC-MS ESI-
NADP	nicotinamide adenine dinucleotide phosphate	HMDB00217	743.0741142	UPLC-MS ESI-
PEP	phosphoenolpyruvic_acid	HMDB00263	166.974516	UPLC-MS ESI-
PCreatine	phosphocreatine	HMDB01511	210.0286901	UPLC-MS ESI-
