# Supplementary material for: T-Allele Carriers of Mono Carboxylate Transporter One Gene Polymorphism rs1049434 Demonstrate Altered Substrate Metabolization during Exhaustive Exercise
Source: Genes (Basel). 2024 Jul 14;15(7):918. doi: 10.3390/genes15070918 (PMC11275951; doi:10.3390/genes15070918)
Supplement: Supplementary file 1 [file genes-15-00918-s001.zip › Table S2 R2.pdf]

Table S2: *Muscle lipids which demonstrated rs1049434 genotype associated differences in the exercise response.* List of lipidic compounds and level (alteration) of their normalized values with one-legged exercise in vastus lateralis muscle in the carriers and non-carriers of the T-allele, i.e. AT/TT and AA genotypes of polymorphism rs1049434. Values in the column 'AT/TT per AA genotype' report the ratio between fold changes with one-legged exercise (i.e. post vs pre) in AT/TT and AA genotypes, respectively. Reported compounds reflect those for which the false discovery rate adjusted probability for an effect (i.e. the q-value) as calculated with statistical analysis of microarrays (SAM) was below a threshold of 5%. Fold changes are accordingly calculated with SAM.

.

				AT/TT genotype				*AA genotype*				*AT/TT per AA genotype*	
name	*class*	*formula*	*mass [u]*	*pre*	*post*	*fold*	*q*	*pre*	*post*	*fold*	*q*	*post vs pre*	*q*
CoA 18:0;O4	acyl CoenzymeA	C39H70N7O21P3S	1097.356	2.39 ± 3.45	1.39 ± 1.44	0.58	63	1.20 ± 1.61	3.27 ± 2.79	2.72	0	0.32 ± 0.69	51
CoA 18:3;O4	acyl CoenzymeA	C39H64N7O21P3S	1091.309	2.45 ± 3.46	2.03 ± 2.71	0.83	78	1.21 ± 1.62	5.45 ± 4.66	4.50	0	0.28 ± 0.64	47
CoA 20:3;O3	acyl CoenzymeA	C41H68N7O20P3S	1103.345	1.86 ± 2.35	1.78 ± 2.32	0.95	78	0.99 ± 1.19	4.61 ± 3.23	4.65	0	0.27 ± 0.62	47
FA 14:4;O4	fatty acid	C14H20O6	284.126	1.63 ± 1.33	1.08 ± 0.68	0.66	52	1.36 ± 2.52	2.12 ± 0.95	1.56	48	0.08 ± 0.07	0
FA 28:0;O2	fatty acid	C28H56O4	456.418	1.14 ± 0.68	0.99 ± 0.93	0.86	63	0.56 ± 0.93	1.41 ± 0.54	2.51	59	0.09 ± 0.07	0
MGDG 60:12	glycerolipid	C69H110O10	1098.810	2.19 ± 2.89	1.31 ± 1.28	0.60	63	1.12 ± 1.42	2.89 ± 2.3	2.59	0	0.30 ± 0.61	47
TG 78:5;O3	glycerolipid	C81H148O9	1265.112	3.00 ± 4.58	1.21 ± 1.22	0.40	30	1.54 ± 2.19	3.48 ± 2.25	2.26	0	0.13 ± 0.25	17
PI O-66:6	glycerophospholipid	C75H137O12P	1260.985	1.65 ± 1.65	1.48 ± 1.08	0.90	63	0.93 ± 0.9	1.85 ± 0.88	2.00	0	0.38 ± 0.37	47
PIP3 36:8;O	glycerophospholipid	C45H74O23P4	1106.357	3.16 ± 4.71	2.18 ± 3.05	0.69	71	1.44 ± 2.06	6.78 ± 6.77	4.72	0	0.27 ± 0.65	47
PIP3 36:9;O	glycerophospholipid	C45H72O23P4	1104.342	2.18 ± 2.91	1.46 ± 1.73	0.67	68	1.16 ± 1.43	3.52 ± 3.08	3.03	0	0.26 ± 0.46	47
PS O-54:4	glycerophospholipid	C60H112NO9P	1021.808	3.17 ± 5.26	1.50 ± 2.07	0.47	55	2.13 ± 2.58	5.20 ± 4.71	2.44	0	0.59 ± 1.3	62
ACer 44:3;O3	sphingolipid	C44H81NO5	703.612	1.73 ± 1.91	1.17 ± 1.41	0.68	67	0.72 ± 0.73	5.06 ± 4.79	7.06	0	0.21 ± 0.46	47
Hex2Cer 46:0;O6	sphingolipid	C58H113NO17	1095.801	1.97 ± 2.66	1.61 ± 2.14	0.82	75	1.09 ± 1.36	4.37 ± 2.84	4.00	0	0.22 ± 0.49	47
IPC 54:3;O5	sphingolipid	C60H114NO14P	1103.798	2.03 ± 2.59	1.24 ± 1.26	0.61	55	1.02 ± 1.25	2.84 ± 1.88	2.77	0	0.27 ± 0.58	47
