# Supplementary material for: T-Allele Carriers of Mono Carboxylate Transporter One Gene Polymorphism rs1049434 Demonstrate Altered Substrate Metabolization during Exhaustive Exercise
Source: Genes (Basel). 2024 Jul 14;15(7):918. doi: 10.3390/genes15070918 (PMC11275951; doi:10.3390/genes15070918)
Supplement: Supplementary file 1 [file genes-15-00918-s001.zip › Table S3 R2.pdf]

Table S3: *Structure-function relationships*. List of (relevant correlations) meeting the criteria of |r| > 0.50, between markers of the slow fiber phenotype (the percentage and area_percentage of slow type muscle fibers), muscle perfusion (capillary density and capillary-to-fiber ratio), and the oxidative phenotype (COX4I1_per_GAPDH_mRNA) of vastus lateralis muscle. Relationships that are of particular interest regarding the hypothesis of the influence of the assessed MCT1 genotype relative to structural hallmark**s**, and passed the threshold of p < 5%, appear in bold.

node 1	*node 2*	*r-value*	*p-value*
**slow_fiber_percentage**	**TAG pre**	**0.585**	**0.036**
**slow fiber percentage**	**TAG post**	**0.604**	**0.049**
slow_fiber_percentage	G6P fold	0.575	0.025
**slow fiber percentage**	**glycogen pre**	**0.592**	**0.026**
**slow_fiber_area_percentage**	**HDL fold**	**-0.628**	**0.022**
**slow_fiber_area_percentage**	**G6P fold**	**0.501**	**0.040**
Capillary density	RER delta 1leg	-0.538	0.087
Capillary density	RER max 2leg	-0.545	0.083
Capillary density	T-Chol post	-0.526	0.036
**Capillary density**	**T-Chol fold**	**-0.589**	**0.021**
**Capillary density**	**LDL fold**	**-0.570**	**0.033**
**Capillary density**	**bHb (blood) fold**	**-0.669**	**0.024**
Capillary density	capillary-to-fiber ratio	0.493	0.014
Capillary-to-fiber ratio	RERmax 2leg	-0.568	0.069
**Capillary- to-fiber ratio**	**bHb (blood) fold**	**-0.604**	**0.049**
Capillary-to-fiber ratio	MCSA slow	0.546	0.019
Capillary-to-fiber ratio	MCSA fast	0.494	0.037
**COX4I1 / GAPDH_mRNA**	**RER max 1leg**	**0.552**	**0.027**
COX4I1 / GAPDH_mRNA	RER max 2leg	0.558	0.074
**COX4I1 / GAPDH_mRNA**	**LDL pre**	**-0.652**	**0.005**
**COX4I1 / GAPDH_mRNA**	**LDL post**	**-0.558**	**0.031**
**COX4I1_/ GAPDH_mRNA**	**HDL fold**	**0.924**	**<0.001**
**COX4I1 / GAPDH_mRNA**	**HDL post**	**0.571**	**0.017**
COX4I1 / GAPDH_mRNA	TAG fold	0.525	0.054
**COX4I1 / GAPDH mRNA**	**glycogen post**	**0.794**	**<0.001**
